# The changing epidemiology and long-term outcomes of patients with CRE infections

**DOI:** 10.1017/ash.2026.10348

**Published:** 2026-04-20

**Authors:** Sunish Shah, Lloyd G. Clarke, Erin K. McCreary, Ellen G. Kline, Emre Yucel, Alexandre H. Watanabe, Ryan K. Shields

**Affiliations:** 1 Division of Infectious Diseases, Department of Medicine, University of Pittsburgh, Pittsburgh, PA, USA; 2 Department of Pharmacy, University of Pittsburgh Medical Center, Pittsburgh, PA, USA; 3 Merck: Merck & Co Inc, USA

## Abstract

**Background:**

The impact of organism species and underlying comorbidities on long-term clinical outcomes for patients with carbapenem-resistant Enterobacterales (CRE) infections is unknown.

**Methods:**

Patients with CRE-positive cultures from 2011 to 2019 were monitored for 1-year. Patients without signs of infection were categorized as colonization. Recurrent cases were defined as isolation of the same CRE species >90 days after the index case.

**Results:**

720 patients met inclusion criteria and accounted for 749 index cases, which decreased over time. The median (range) age was 61 (20–97) years, 53% (397/749) were male, 20% (151/749) received solid organ transplant (SOT), and 44% resided in the ICU at the time of CRE isolation. The colonization rate was 34% (257/749). Pneumonia and bacteremia represented the most common infection types accounting for 25% (185/749) and 13% (95/749) of all cases, respectively. *Klebsiella pneumoniae* was most common pathogen (58%), followed by *Enterobacter cloacae complex* (23%), and *Escherichia coli* (10%). Of the 554 sequenced isolates, 35% (195/554) were KPC-2, 32% (175/554) KPC-3, and 32% (177/554) non-KPC-producing. This changed over time where KPC-2-producing CRE were most prevalent in 2011, KPC-3 most prevalent between 2015–2017, and non-KPC from 2018–2019. Among 90-day survivors, the CRE recurrence rate was 21% (108/513). Charlson comorbidity index (OR: 1.11; 95% CI: 1.05–1.18; *P* < .001), isolation of a prior CR pathogen (OR: 2.49; 95% CI 1.32–4.72; *P* = .005), and ICU admission (OR: 3.35; 95% CI 2.14–5.24; *P* < .001) were independently associated with 90-day mortality, while SOT was associated with lower 90-day mortality (OR: 0.55; 95% CI 0.32–0.94; *P* = .029). Overall, 90-day mortality rates were lower among patients with CRE-positive cultures between years 2016 – 2019 (24% [63/262]) compared to years 2011 – 2015 (35.5% [173/487]; *P* = .001).

**Conclusion:**

CRE are increasingly diverse and associated with significant morbidity and healthcare utilization across varying patient groups. Long-term survival of patients infected with CRE has improved over time while overall incidence of CRE has decreased.

## Introduction

Carbapenem-resistant *Enterobacterales* (CRE) are categorized by the World Health Organization as a critical priority for research and development of new antibiotics to combat the threat posed to public health.^1^ Despite entry of several new antibiotics into the global market, the outcomes of patients infected by CRE remain suboptimal.^2–5^ Slow uptake of novel therapies, limited access to molecular diagnostics, and clinician education have all played a role in delaying progress.^6^ A key factor complicating selection of optimal antibiotic therapy is the rapidly changing epidemiology of CRE infections.^2,7^ In the United States, production of *Klebsiella pneumoniae* carbapenemases (KPC) predominated as the main cause of carbapenem resistance for most of the 2000s,^8^ prompting the development of novel *β*-lactamase inhibitors (BLI) avibactam, relebactam, and vaborbactam capable of inhibiting KPC-mediated *β*-lactam hydrolysis. More recent investigations, however, suggest the molecular epidemiology of CRE is increasingly diverse.^2,7^ Indeed, carbapenemase- (CP) and non-carbapenemase-producing (non-CP) CRE are identified with nearly equal frequency, and the prevalence of metallo-*β*-lactamases and oxacillinase (OXA)-48-like carbapenemases is increasing.^9^ Thus, treating CRE infections is dependent upon a deep understanding of the regional and local epidemiology of CRE, access to molecular tools capable of differentiating types of carbapenem resistance, and the availability of antibiotic susceptibility testing of and access to CRE-targeted antibiotics.

Clinical outcomes of patients infected by diverse CRE species, including non-CP CRE, have not been well established. As a result, treatment regimens vary considerably, and long-term outcomes are unknonwn.^2^ Given the shifting epidemiology, it is unlikely that any single agent will be effective across diverse CRE pathogens and infection types. More practically, each of the newly approved CRE-active agents may offer a specific therapeutic niche.^10^ Defining such niches is dependent upon future comparative-effectiveness studies that include robust secondary outcomes like healthcare utilization, recurrent infections, long-term efficacy, and treatment-emergent resistance. Moreover, understanding which patients are prone to deleterious outcomes is critical to strategize aggressive countermeasures, including use of novel antibiotics empirically and in combination. To address some of these critical knowledge gaps related to changing CRE epidemiology over time and long-term clinical outcomes, we conducted a longitudinal, observational study of patients from whom CRE was isolated over a 9-year period. Patients were included before and after the availability of ceftazidime-avibactam and meropenem-vaborbactam to establish a benchmark for long-term outcomes.

## Methods

### Patient selection

This was a single-center, retrospective cohort study of patients with CRE-positive clinical cultures. Patients were included at the time of their first CRE-positive culture from 2011 to 2019 according to contemporary carbapenem breakpoints as defined by the Clinical and Laboratory Standards Institute.^11^ Interpretive criteria were applied retrospectively throughout the study period for consistency in case definitions as we previously reported.^12^ Isolates with intermediate carbapenem susceptibility were excluded, as were peri-rectal screening or other surveillance cultures growing CRE. Index cases were defined as the first isolation of a unique CRE species. Recurrent cases were defined as isolation of the same CRE species after at least 90 days. Patients were re-included if a unique CRE species was subsequently isolated after the index case. Infection types were defined according to standardized NHSN criteria.^13^ Patients who did not meet criteria for infection were considered to be colonized.

### Whole-genome sequencing

Available CRE isolates underwent whole-genome sequencing on the Illumina platform as previously reported.^14^ Sequences were assembled with SPAdes,^15^ and annotated with prokka.^16^ All CRE species were determined by GTDB-Tk, and sequence types (ST) were defined by standard criteria (https://github.com/tseemann/mlst).^17^ Resistance genes were analyzed through ResFinder, AMRFinderPlus, and Kleborate.^18–22^ All genomes are publicly available; NCBI accession numbers are listed in the Supplementary Table.

### Statistical analysis

Comparisons across CRE species were made by using descriptive statistics. Categorical or continuous variables associated with 90-day mortality were compared by χ^2^ or Mann–Whitney tests, respectively. Multivariate analyses were performed with a stepwise multivariate logistic regression model including factors that demonstrated a *P*-value < .10 on univariate analysis. To compare species-specific differences, patients were analyzed at the time of first isolation of CRE by each species.

## Results

### Patient demographics and clinical characteristics

Of the 1,049 CRE-positive index cases identified, 300 were excluded due to phenotypes that were determined to be inconsistent with CRE. The remaining 749 index cases were identified among 720 unique patients, including 7 patients with >1 CRE species at the time of initial culture and 28 patients with positive cultures for different CRE species on separate occasions (Table 1). Overall, the median (range) age was 61 (20–97) years, 53% (397/749) were male, and 20% (151/749) were solid organ transplant (SOT) recipients. The specific types of organ transplant included heart (n = 6), liver (n = 38), lung (n = 58), multivisceral (n = 14), kidney (n = 18), and small bowel/pancreas transplant (n = 17). The median (range) Charlson Comorbidity Index was 5 (0–22), and 50% (375/749) of patients were admitted to the hospital from their home. At the time of CRE isolation, 44% (326/749) of patients resided in the intensive care unit (ICU).

**Table 1.** Patient demographics, clinical characteristics, and key outcomes across CRE species

Patient characteristics	Kp (N = 427)	Enc (N = 170)	Ec (N = 77)	Ka (N = 44)	Sm (N = 24)	Total^a^ (N = 749)
Median age in years (range)	60 (20–97)	62 (21–95)	61 (20–95)	62 (21–84)	62 (26–83)	61 (20–97)
Male sex, n (%)	220 (52)	91 (54)	40 (52)	26 (59)	18 (75)	397 (53)
Median Charlson score (range)	5 (0–15)	6 (0–22)	6 (0–15)	5 (1–16)	5.5 (0–12)	5 (0–22)
SOT, n (%)	98 (23)	30 (18)	14 (18)	5 (11)	3 (13)	151 (20)
Other immunocompromising condition, n (%)	36 (8)	11 (6)	9 (12)	4 (9)	1 (4)	61 (8)
Prior CR pathogen, n (%)^b^	49 (11)	10 (6)	7 (9)	5 (11)	6 (25)	78 (10)
Admission source, n (%)						
Outpatient clinic	4 (1)	4 (2)	1 (1)	0 (0)	0 (0)	9 (1)
Home	211 (49)	84 (49)	40 (52)	25 (57)	12 (50)	375 (50)
LTAC/SNF	84 (20)	22 (13)	15 (19)	7 (16)	2 (8)	130 (17)
Outside hospital	120 (28)	54 (32)	18 (23)	9 (20)	9 (38)	213 (28)
Inpatient rehab	8 (2)	6 (4)	3 (4)	3 (7)	1 (4)	22 (3)
ICU residence, n (%)	192 (45)	70 (41)	29 (38)	21 (48)	10 (42)	326 (44)
Infection Type, n (%)						
Blood	59 (14)	27 (16)	4 (5)	0 (0)	4 (17)	95 (13)
Pneumonia	102 (24)	47 (28)	15 (19)	11 (25)	9 (38)	185 (25)
Intrabdominal	32 (7)	24 (14)	12 (16)	8 (18)	0 (0)	78 (10)
Colonized	172 (40)	35 (21)	24 (31)	19 (43)	5 (21)	257 (34)
UTI	36 (8)	19 (11)	12 (16)	2 (5)	1 (4)	71 (9)
SSTI	10 (2)	8 (5)	1 (1)	1 (2)	2 (8)	22 (3)
Other	16 (4)	10 (6)	9 (12)	3 (7)	3 (13)	41 (5)
Median LOS in days (IQR)	29 (11–64)	34 (12–70)	32 (7–92)	31 (13–61)	32 (10–83)	31 (11–69)
Discharge disposition, n (%)						
Death/hospice	90 (21)	40 (23.5)	17 (22)	13 (30)	4 (17)	164 (22)
Home/self-care	116 (27)	43 (25)	26 (34)	7 (16)	7 (29)	204 (27)
LTAC/SNF	144 (34)	49 (29)	22 (29)	14 (32)	9 (38)	239 (32)
Transfer to another hospital	19 (4)	12 (7.5)	2 (3)	2 (5)	0 (0)	35 (5)
Inpatient rehab	58 (14)	26 (15)	10 (13)	8 (18)	4 (17)	107 (14)
30-day re-admission, n (%)^c^	60 (18)	21 (16)	5 (8)	5 (16)	1 (5)	92 (16)
90-day re-admission, n (%)^c^	127 (38)	34 (26)	18 (30)	9 (29)	5 (25)	195 (33)
CRE recurrence, n (%)†	101 (34)	3 (3)	0 (0)	1 (4)	3 (17)	108 (21)
365-day readmissions, n(%)^c^	210 (62)	49 (38)	34 (57)	15 (48)	10 (50)	324 (55)
Median admissions (range)	2 (1–13)	2 (1–11)	2 (1–10)	2 (1–8)	3 (1–5)	2 (1–13)
Median hosp. days (range)	43 (3–245)	44 (3–253)	42 (1–245)	53 (1–170)	39 (23–175)	43 (1–253)
30-day mortality, n (%)	88 (21)	35 (21)	10 (13)	14 (32)	3 (13)	150 (20)
90-day mortality, n (%)	131 (31)	53 (31)	25 (32)	20 (45)	6 (25)	236 (32)
365-day mortality, n (%)	197 (46)	76 (45)	34 (44)	23 (52)	12 (50)	344 (46)

a Seven patients were initially infected with >1 CRE species; ^b^Includes carbapenem-resistant *Acinetobacter baumannii* or *Pseudomonas aeruginosa* from patients with prior colonization or infection; ^c^Among patients surviving hospital discharge; †Among patients surviving 90 days.

Kp, *Klebsiella pneumoniae*; Enc, *Enterobacter cloacae* complex; Ec, *Escherichia coli;* Ka, *Klebsiella aerogenes;* Sm, *Serratia marcescens;* ICU, intensive care unit; SOT, solid organ transplant; LTAC, long-term acute care facility; SNF, skilled nursing facility; IQR, interquartile range; LOS, length of stay; CR, carbapenem-resistant; UTI, urinary tract infection; SSTI, skin/soft tissue infection; CRE, carbapenem-resistant Enterobacterales; hosp, hospital.

**Figure 1.**
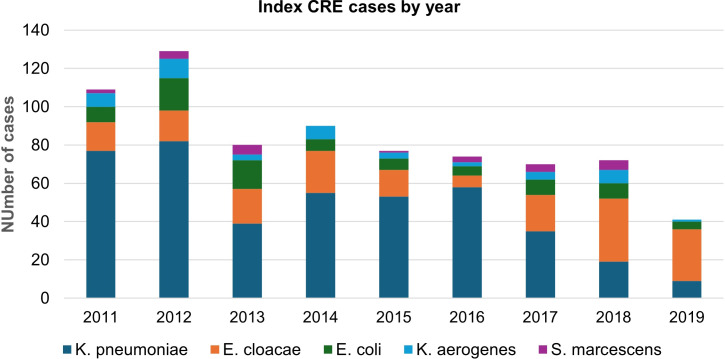
A total of 720 unique patients were included in the analysis who accounted for 749 unique CRE index cases. Cases are defined per patient per species. Seven patients were infected with 2 CRE species at the time of first isolation. An additional 28 patients accounted for more than 1 CRE case due to different species isolated on separate occasions.

*Klebsiella pneumoniae* was the most common CRE species identified, accounting for 57% (427/749) of index cases. *Enterobacter cloacae, Escherichia coli, Klebsiella aerogenes,* and *Serratia marcescens* accounted for 23% (170/749), 10% (77/749), 6% (44/749), and 3% (24/749) of cases, respectively. The number of CRE index cases decreased over time, which was associated with a decreased number of *K. pneumoniae* cases specifically (Figure 1). The overall colonization rate was 34% (257/749), which varied from 43% (19/44) for *K. aerogenes* to 21% (35/170) for *E. cloacae*. Among patients with infection, pneumonia and bacteremia represented the most common infection types, accounting for 38% (185/492) and 19% (95/492) of patients, respectively. Types of infection also varied by CRE species wherein the proportion of cases with pneumonia was highest for *S. marcescens* (38%, 9/24) and lowest for *E. coli* (19%, 15/77), but the opposite for urinary tract infections, which were more common for *E. coli* (16%, 12/77) than *S. marcescens* (4%, 1/24) or *K. aerogenes* (5%, 2/44).

### Clinical outcomes

**Figure 2.**
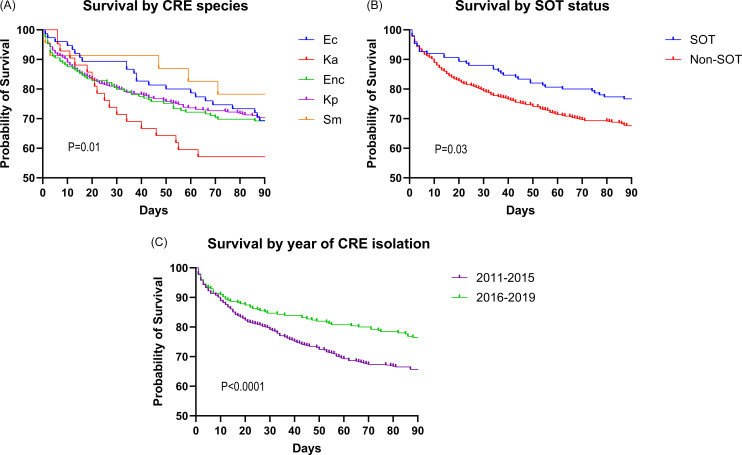
The infection rate for patients with CRE-positive cultures between 2011–2015 was 63% (309/487) compared to 69% (183/262) between 2016–2019 (*P* = .079). The corresponding mortality rates for patients were 35.5% (173/487) and 24% (63/262), respectively (*P* = .001). CRE, carbapenem-resistant Enterobacterales; SOT, solid organ transplant; Kp, *Klebsiella pneumoniae*; Enc, *Enterobacter cloacae* complex; Ec, *Escherichia coli;* Ka, *Klebsiella aerogenes;* Sm, *Serratia marcescens*.

The overall 30-, 90-, and 365-day mortality rates were 20% (150/749), 32% (236/749), and 46% (344/749), respectively. Mortality rates varied significantly by CRE species (Figure 2A). Specifically, patients with *K. aerogenes* experienced the highest rate of death by 90 days at 45% (20/44) compared to patients with *S. marcescens* at 25% (6/25). Patients with a history of SOT and those with index cases between 2016–2019 exhibited lower rates of death compared to non-SOT and cases identified between 2011–2015, respectively (Figures 2B and 2C). The infection rate of patients between 2011–2015 was 63% (309/487) compared to 69% (183/262) in 2019–2019 (*P* = .079). Older age (>60 yr; 35% vs 23%; *P* < .0001) and ICU residence (46% vs 17%; *P* < .0001) were associated with higher 90-day mortality on univariate analysis (Table 2). In a stepwise multivariate logistic regression model, Charlson comorbidity index (OR: 1.11; 95% CI: 1.05–1.18; *P* < .001), isolation of a prior CR pathogen (OR: 2.49; 95% CI 1.32–4.72; *P* = .005), and ICU residence (OR: 3.35; 95% CI 2.14–5.24; *P* < .001) were independently associated with 90-day mortality, while SOT was associated with lower 90-day mortality (OR: 0.55; 95% CI 0.32–0.94; *P* = .029).

**Table 2.** Univariate and multivariate analysis of factors associated with 90-day mortality among CRE index cases

Patient characteristics	Non-survivors (n = 236)	Survivors (n = 513)	Univariate OR (95% CI)	Multivariate OR (95% CI)
Median age in years (range)	63 (21–97)	59 (20–96)	1.02 (1.01–1.03)	1.01 (0.99–1.03)
Male sex, n (%)	137 (42)	260 (51)	1.34 (0.98–1.84)	1.26 (0.83-1.89)
**Median Charlson score (range)**	**6 (0–20)**	**5 (0–22)**	**1.11 (1.07–1.17)**	**1.09 (1.02–1.16)**
**SOT, n (%)**	**36 (15)**	**115 (22)**	**0.62 (0.41–0.93)**	**0.50 (0.29–0.85)**
Other immunocompromising condition, n (%)	19 (8)	42 (8)	0.98 (0.56–1.73)	
**Prior CR pathogen, n (%)**	**32 (14)**	**46 (9)**	**1.59 (0.99–2.57)**	**2.31 (1.24–4.31)**
ICU residence, n (%)	155 (66)	171 (33)	3.83 (2.76–5.30)	3.38 (2.25–5.08)
Pathogen, n (%)^a^	N = 235	N = 507		
*E. coli*	25 (11)	52 (10)	Reference	
*E. cloacae* complex	53 (23)	117 (23)	0.93 (0.52–1.63)	
*K. pneumoniae*	131 (56)	296 (58)	0.92 (0.55–1.53)	
*K. aerogenes*	20 (9)	24 (5)	1.60 (0.76–3.37)	
*Serratia species*	6 (3)	18 (4)	0.69 (0.25–1.95)	
Infection type, n (%)^b^	N = 178	N = 314		0.97 (0.623-1.51)
Blood	33 (19)	62 (20)	1.09 (0.41–2.89)	
Pneumonia	79 (44)	106 (34)	1.49 (0.61–3.66)	
Intrabdominal	30 (17)	48 (15)	1.22 (0.46–3.21)	
UTI	16 (9)	55 (18)	0.59 (0.21–1.64)	
SSTI	6 (3)	16 (5)	0.75 (0.21–2.66)	
Other	14 (8)	27 (9)	1.00 (0.35–2.90)	

a Excluding 7 patients infected with >1 CRE species at the time of index isolation; ^b^Excluding 257 cases that were deemed to be CRE colonization.

Note. Independent predictors of survival are denoted in boldface font.

ICU, intensive care unit; SOT, solid organ transplant; CR, carbapenem-resistant; SSTI, skin/soft tissue infection; UTI, urinary tract infection; CRE, carbapenem-resistant Enterobacterales; OR, odds ratio; CI, confidence interval.

The median (interquartile range) LOS for CRE index cases was 31 (11–69) days, and 22% (164/749) of patients did not survive hospital discharge. Among those who did survive initial discharge (n = 585 cases), the rate of 30- and 90-day re-admission was 16% (92/585) and 33% (195/585), respectively. Over the 1-year following discharge, 55% (324/585) were re-admitted to the hospital for a median (range) of 2 (1–13) admissions accounting for a median total of 43 additional days of hospitalization. Among patients who survived at least 90 days from CRE index case (n = 513 cases), rates of recurrence were significantly higher for those with *K. pneumoniae* (34%, 101/296) compared to all other CRE species (3%, 7/218; *P* < .001).

### Molecular characteristics of CRE isolates

**Figure 3.**
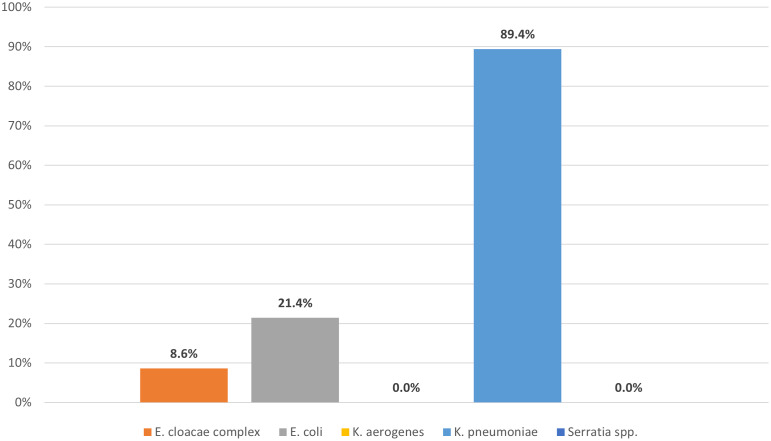
A total of 554 isolates underwent whole-genome sequence analysis, including *E. cloacae* (n = 93), *E. coli* (n = 28), *K. aerogenes* (n = 15), *K. pneumoniae* (n = 407), and *Serratia species* (n = 11).

Combining index cases (n = 749) and recurrence cases (n = 108), a total of 857 CRE cases were included in the analysis. Of these, CRE isolates were available in 64% (554/857) from archived biospecimens, and were subsequently confirmed to be the same CRE species by WGS analysis as reported in the patient’s medical record. *K. pneumoniae* accounted for 75% (407/554) of sequenced isolates, followed by *E. cloacae* complex (17%, 93/554), *E. coli* (5%, 28/554), *K. aerogenes* (3%, 15/554), and *Serratia spp.*(2%, 11/554). The rate of KPC-producing CRE varied from 89% (364/407) among *K. pneumoniae* to 0% among *K. aerogenes* and *Serratia* spp (Figure 3). Among *K. pneumoniae* specifically, KPC-2 and KPC-3 variants were identified in 51% (187/364) and 46% (169/364), respectively. ST258 accounted for 93% (339/364) of all KPC-producing *K. pneumoniae* isolates. The presence of non-carbapenemase *β*-lactamases is summarized in Supplementary Table 1. Overall, 89% (361/407) and 18% (72/407) of *K. pneumoniae* isolates harbored mutations in *ompK35* and *ompK36,* respectively. The rate of *ompK36* mutations was lower among isolates with KPC-3 (3%, 5/169) compared to KPC-2 (26%, 49/187; *P* < .001) or non-KPC (42%, 18/43; *P* < .001) *K. pneumoniae*. Among the 427 patients with *K. pneumoniae,* 324 isolates underwent WGS analysis, including KPC-2 (n = 146), KPC-3 (n = 135), non-CP (n = 41), and other KPC variants (n = 2). The 90-day mortality rate among patients with non-CP, KPC-2, and KPC-3 producing *K. pneumoniae* was 34% (14/41), 38% (55/146), and 24% (32/135), respectively.

Among *E. cloacae* complex isolates (n = 93), 9% (8/93) harbored KPC and 6% harbored CTX-M-15 (6/93), but most isolates did not harbor acquired *β*-lactamases. In contrast, 89% (25/28) of *E. coli* isolates contained plasmid-acquired, serine *β*-lactamases, including KPC (n = 6), CTX-M-15 (n = 12), and others (n = 7).

Overall, WGS data were available from 471 of the 749 index CRE cases, among which 63% (298/471) of isolates harbored KPC and 37% (173/471) did not. Overall rates of 30-day mortality were 21% (62/298) and 22% (38/173) among cases initially infected with KPC- or non-KPC-producing CRE, respectively.

## Discussion

Our study describes a rapidly changing CRE epidemiology from 2011 to 2019 at a major academic medical center in the United States, and is one of the first to report both short- and long-term outcomes following the first year after isolation of CRE among hospitalized patients.^12^ We found that survival improved over time, but the total burden of disease, as measured by recurrent infections and hospital re-admissions, remained significant. In fact, only a minority of patients who survived hospital discharge were discharged home, underscoring the added burden placed on long-term care facilities and nursing homes.^23^ We also identified a number of factors associated with improved clinical outcomes, including the year of treatment, pathogen type, and receipt of SOT prior to CRE infection. Although some of these factors may be expected, others were not, and attest to the need for continued clinical and molecular surveillance of CRE infections over time. Such data can be used to strategize treatment approaches in the current era of widely available *β*-lactam/*β*-lactamase inhibitor (BL/BLI) agents with in vitro activity against KPC and use of rapid molecular diagnostic tests.

Not surprisingly during this period, Most cases were due to ST258 KPC-producing *K. pneumoniae;* ^2,7,23^ however, the increasing frequency of non-CP CRE is particularly notable because these isolates are genetically diverse. Data collected across 49 hospitals showed that clinical outcomes of patients infected with non-CP CRE are similar to those infected with KPC-producing CRE.^2^ These data, including those in the current report, contrast a prior single-center report of cases from 2013–2016 that identified a 4-fold increase in 14-day mortality rates among patients infected with CP- versus non-CP CRE.^24^ Another key findings is a high proportion of CRE cases (28.6%; 300/1,049) that were unlikely to be reliably carbapenem-resistant based on the reported phenotype, corroborating a prior multicenter report.^2^ Our approach in adjudicating CRE index cases was done through manual inspection of each phenotypic profile wherein standardized criteria were applied. Cases were excluded if carbapenem resistance was identified, but no other resistance to *β*-lactams, or if ampicillin and cefazolin were reported as susceptible. Although a more judicious approach would include repeat susceptibility testing on these isolates, this could not be done retrospectively. Notably, in a study of 1,040 CRE cases, 22% (228/1,040) of isolates were categorized as unconfirmed CRE after repeat testing in a central laboratory failed to identify carbapenem resistance.^2^ Taken together, these data indicate that routine testing may misclassify a significant percentage of cases as CRE, particularly among non-CP CRE. Further studies are needed to confirm these findings prospectively with repeat testing in real time, exclusion of mixed cultures, and molecular investigation of the underlying mechanisms of resistance.

The increasing proportion of non-CP CRE at our center coincided with a decline in the number of KPC-producing CRE cases over time. This trend is consistent with a large epidemiological study across the Veterans Affairs healthcare system during the same period.^25^ Interestingly, multiple distinct ST258 sublineages were identified and associated with device- and ward-level outbreaks,^26^ but a discernable shift occurred between 2014 and 2015 from clade 1, KPC-2-producing *K. pneumoniae* to clade 2, KPC-3-producing *K. pneumoniae.* Although this is aligned with the availability of ceftazidime-avibactam in the United States in 2015, the clade 2, KPC-3 producing sublineage emerged prior to clinical use at our center.^26^ It is more likely that adaptive mutations, including those in *pfeA* and *rcsA*, arose overtime allowing clade II to persist at our center. This persistence, however, was short-lived as rates of KPC-producing *K. pneumoniae* declined in 2017, a trend that is mirrored across the United States and globally.^27,28^ The reduction in ST258 KPC-producing *K. pneumoniae* was in fact a driving factor associated with a significantly lower rate CRE index cases by the end of the study period. To date, it is unclear why rates of KPC-producing CRE declined so precipitously across the United States, but notably similar trends have been identified among ST11 in China and other STs globally.^27^ It is possible that interventions such as aggressive infection control, increased laboratory recognition of KPC, improved stewardship practices, and introduction of several novel BL/BLI agents with in vitro activity against KPC collectively affected these rates.^29^ Interestingly, rates of CP CRE have continued to decline in surveillance studies conducted since 2019, but as the proportion of KPC has declined, the rates of OXA-48 and NDM have slowly risen.^9^ These data attest to the continued need for active surveillance studies to understand and adapt to continuing shifts in the CRE epidemiology, including the expanded use of whole-genome sequencing to identify clonal relatedness and expansion of new clones over time.

Next, our data confirm that overall outcomes for patients with CRE infections have improved over time.^12^ Such changes can be clearly ascribed to improved treatment with BL/BLI agents rather than older agents like aminoglycosides and colistin.^30,31^ Molecular diagnostics have also improved detection of KPC, prompting earlier active therapy.^32^ Although current guidelines recommend use of meropenem-vaborbactam, ceftazidime-avibactam, or imipenem-relebactam for the treatment of KPC-producing CRE, there is limited evidence to prioritize one agent over another. Comparative-effectiveness data for meropenem-vaborbactam and ceftazidime-avibactam is limited to a single retrospective cohort study of 131 patients with CRE infections where no significant mortality differences were identified.^33^ It may be postulated that meropenem-vaborbactam should be preferred over ceftazidime-avibactam for cases of pneumonia considering pneumonia has been shown to be an independent predictor of failure for patients receiving ceftazidime-avibactam for CRE infections and the bronchial epithelial lining fluid exposure of ceftazidime-avibactam is only ∼30% of serum concentrations.^34,35^ Nevertheless, future studies are needed, including those that evaluate robust long-term outcomes using the data described in this study as a key benchmark.

Mortality rates were lower among SOT patients with CRE in our study compared to non-SOT patients (Figure 2 **)**. These data extend our prior investigations and are consistent with case-control studies.^12,36^ In a propensity score-adjusted analysis of patients with bacteremia, 28-day and 90-day mortality rates were significantly lower for SOT recipients compared with non-SOT patients.^36^ In a separate analysis of patients with CRE infection specifically, SOT were shown to have a 7% lower risk 30-day mortality after inverse probability weighted adjustment.^37^ Collectively the data suggest that immunosuppression, including use of corticosteroids, mitigates an inflammatory response in the setting of acute bacterial infections. It is also possible that SOT may have enhanced provider vigilance and improved access to health care. Furthermore, it is likely that clinicians exhibit lower thresholds to treat SOT patients when there are not clear signs of infection, which may portend improved longer-term outcomes.

When outcomes were stratified by CRE organism, we found the highest mortality rates with *K. aerogenes* compared to other species (Figure 2). Perhaps more importantly, our overall findings show that infection types, severity of illness, and clinical outcomes vary by CRE species, indicating that further investigations are needed to assess pathogen virulence, propensity for recurrent infections, and ultimately evolution of resistance to frontline therapies. To this end, it is clear that CRE encompass a wide range of pathogens, and further discrimination of the appropriate terminology is needed in medical education and treatment pathways.^38^

While the strengths of this study include its large sample size across a decade, standardized definitions of carbapenem resistance, and our ability to track patients within the same healthcare system over time, several limitations should be recognized. First, this study was performed retrospectively, and thus, the availability of clinical isolates was based on specimens that had been previously archived, and our determination of infection types was subject to documentation in the electronic health record. We were also unable to evaluate improved infection prevention practices in real time; however, outbreak detection and associated surveillance changes at our center have been previously reported.^26^ Secondly, this study is an epidemiology study and specific treatment regimens were not assessed, but is a focus on future investigations. Third, our study period ended prior to the COVID-19 pandemic; however, contemporary data suggests that trends for lower rates of KPC isolation continued postpandemic.^9,27^ Finally, this study was single center within the United States, and therefore external validity may be limited when extrapolating these results outside of the United States where epidemiologic and treatment patterns vary. Nevertheless, our data have unveiled several unique features of CRE infections, including a rapidly changing epidemiology and hypothesis-generating findings for patients with varying underlying diseases where long-term outcomes and healthcare utilization can be optimized.

## Supporting information

10.1017/ash.2026.10348.sm001 Shah et al. supplementary material Shah et al. supplementary material

## Supplementary material

The supplementary material for this article can be found at https://doi.org/10.1017/ash.2026.10348.
